# Expanded range of *Haemagogus leucocelaenus* in yellow fever hotspots: new findings from Santa Catarina State, southern Brazil

**DOI:** 10.1590/0074-02760240240

**Published:** 2025-07-04

**Authors:** Sabrina Fernandes Cardoso, Iara Carolini Pinheiro, Larissa Akemi Oliveira Kikuti, Andre Akira Gonzaga Yoshikawa, André Nóbrega Pitaluga, Luísa Damazio P Rona

**Affiliations:** 1 Universidade Federal de Santa Catarina, Departamento de Biologia Celular, Embriologia e Genética, Florianópolis, SC, Brasil; 2 Secretaria da Saúde do Estado de Santa Catarina, Diretoria de Vigilância Epidemiológica, Florianópolis, SC, Brasil; 3 Fundação Oswaldo Cruz-Fiocruz, Instituto Oswaldo Cruz, Rio de Janeiro, RJ, Brasil; 4 Conselho Nacional de Desenvolvimento Científico e Tecnológico, Instituto Nacional de Ciência, Tecnologia e Inovação em Entomologia Molecular, Rio de Janeiro, RJ, Brasil

**Keywords:** yellow fever, Haemagogus, Haemagogus leucocelaenus, mosquitoes

## Abstract

**BACKGROUND:**

The *Haemagogus* genus includes nine mosquito species reported in Brazil, each with distinct distribution patterns. *Haemagogus leucocelaenus*, a major yellow fever vector, is widely distributed throughout the country, while *Haemagogus leucophoebus*, a morphologically similar species, has only been identified in Acre State.

**OBJECTIVES:**

This study evaluated the presence of *Haemagogus* species in southern Brazil by comparing their morphological and molecular characteristics.

**METHODS:**

Mosquitoes were collected from five municipalities in southern Santa Catarina State, Brazil. Each specimen was identified morphologically and photographed. Genomic DNA was extracted, and a Cytochrome C Oxidase Subunit I (COI) gene fragment was amplified using polymerase chain reaction (PCR). The positive amplicons were sequenced for molecular identification.

**FINDINGS:**

New records of *Hg. leucocelaenus* were found in Santa Rosa de Lima, Rio Fortuna, Braço do Norte, São Martinho, and Pedras Grandes, located at the southern edge of the Atlantic Forest. This study expands the known distribution of *Hg*. *leucocelaenus*, the only *Haemagogus* species identified in the area, with 91 specimens collected. Although some specimens exhibited morphological variations that might lead to misidentification as *Hg. leucophoebus*, molecular identification confirmed that all were *Hg. leucocelaenus*.

**MAIN CONCLUSIONS:**

This study is the first to report *Hg. leucocelaenus* in Santa Catarina, Brazil, and provides DNA barcoding sequences from southern Brazil. This method offers a reliable alternative for species identification, especially when combined with morphological analysis. Further molecular studies are needed to determine whether the morphological variations observed indicate intraspecific differences.

Yellow fever (YF) is a non-contagious disease caused by the YF virus (YFV), which belongs to the species *Orthoflavivirus flavi* ^1^ ^,^ ^2^ within the *Orthoflavivirus* genus of the *Flaviviridae* family.^2^ In Brazil, YF is transmitted through two cycles: (i) the sylvatic (or jungle) cycle, involving non-human primates (NHP) and mosquitoes, primarily *Sabethes* spp. (Robineau-Desvoidy, 1827) and *Haemagogus* spp. (Williston, 1896); and (ii) the urban cycle, which has not been reported in Brazil since 1942,^3^ in which YFV is transmitted to humans by *Aedes* sp. (Meigen, 1818) mosquitoes.

Mosquitoes of the *Haemagogus* genus, key vectors of YFV and other arboviruses,^4^ are acrodendrophylic, diurnal, and commonly inhabit forested areas.^5^ They typically breed in natural containers such as tree holes, bamboo internodes, bromeliads, and coconut husks.^6^ This genus consists of 28 species, divided into two subgenera: *Conopostegus* (Dyar, 1925) and *Haemagogus* (Williston, 1896), with a wide distribution across the Americas.^7^ ^,^ ^8^ ^,^ ^5^ In Brazil, nine *Haemagogus* species have been reported, including *Haemagogus leucocelaenus* (Dyar & Shannon, 1924) and *Haemagogus leucophoebus* (Galindo, Carpenter & Trapido, 1953) (from the *Conopostegus* subgenera), as well as *Haemagogus janthinomys* (Dyar, 1921), *Haemagogus tropicalis* (Cerqueira & Antunes, 1938), *Haemagogus spegazzinii* (Brèthes, 1912), *Haemagogus baresi* (Cerqueira, 1960), *Haemagogus capricornii* (Lutz, 1904), *Haemagogus albomaculatus* (Theobald, 1903), and *Haemagogus celeste* (Dyar & Nuñez Tovar, 1927),^9^ all of which belong to the *Haemagogus* subgenera.^10^

*Haemagogus leucocelaenus*, an important vector of YFV in Brazil,^4^ is found throughout the country, from the northern to the southern regions.^10^ This species is morphologically similar to *Hg. leucophoebus*,^11^ whose only recorded occurrence dates back to the 1950s in Acre State.^11^ ^,^ ^12^ ^,^ ^13^ The scarce documentation of *Hg. leucophoebus* in Brazil may be due to challenges in its morphological identification. Females of *Hg*. *leucophoebus* and *Hg. leucocelaenus* are very similar, and according to the main taxonomic keys by Forattini^6^ and Consoli & Lourenço-de-Oliveira,^10^ they can only be distinguished by a few specific features on the head, such as setae. As a result, relying solely on morphology for accurate identification can be challenging. To address this issue, this study aimed to (I) assess the presence of *Haemagogus* mosquitoes in Santa Catarina, Brazil, given that *Hg. leucocelaenus*, a significant vector of YFV, has never been collected in the state,^14^ and (II) characterise these specimens using Cytochrome C Oxidase Subunit I (COI) sequences.

## MATERIALS AND METHODS

**Fig. 1:**
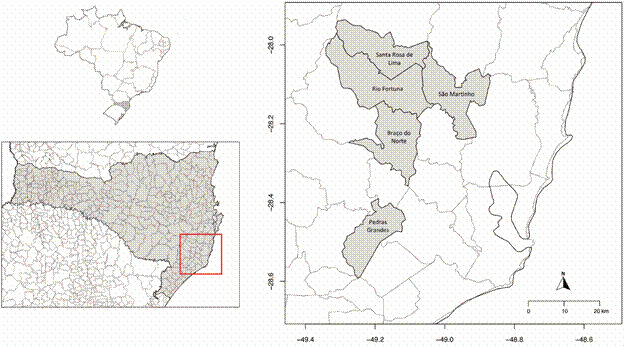
collection sites for *Haemagogus* mosquitoes in Santa Catarina, Brazil. In the upper left, a map of Brazil shows the State of Santa Catarina (SC) highlighted in grey. Below it, a magnified view of SC is presented. On the right, a zoomed-in section of the SC map (marked by a red box) highlights the specific sample collection sites, which are indicated in grey. The x-axis and y-axis of this zoomed-in map represent longitude and latitude, respectively. The maps were created using the *sf*, *maps*, and *mapdata* packages^26^ ^,^ ^27^ ^,^ ^28^ ^,^ ^29^ in R Software, version 4.3.1.^30^

*Entomological collection and morphological identification* - The study was conducted in forested ecosystems within a microregion of southern Santa Catarina State, Brazil (Fig. 1). The detection of YFV in this region by the Directorate of Epidemiological Surveillance in February 2021^15^ prompted subsequent entomological collections. Sampling sites were selected based on the geographical presence of non-human primates diagnosed with YFV. The chosen locations were Santa Rosa de Lima (-28.032639, -49.149684), Rio Fortuna (-28.141177, -49.146304), Braço do Norte (-28.195246, -49.136690), São Martinho (-28.127728, -49.051721), and Pedras Grandes (-28.514485, -49.242145) (Fig. 1).

Collections took place during the summer months of 2023, in January and February. Sampling occurred between 08:00 and 17:00 over three consecutive days in each municipality. Mosquitoes were captured in the canopy and at ground level using a manual collection net and an oral aspirator with protected human attraction. Additionally, four CDC light traps (CDC-LT), powered by carbon dioxide (dry ice), were set up in the canopy.

The captured insects were preserved on dry ice during transport to the laboratory, where they were stored in cryotubes at -80ºC. *Haemagogus* specimens were morphologically identified using a stereomicroscope (SZX16 Olympus) on a cold surface, following the taxonomic keys of Consoli and Lourenço-de-Oliveira^10^ and Forattinni.^6^

*Ethics considerations* - All members of the research team had been vaccinated against YFV before the study. Additionally, to reduce the risk of exposure to pathogens, the field team wore personal protective equipment, including long-sleeved clothing, caps, and boots.

*Molecular analysis* - Molecular identification was performed on 17 *Hg. leucocelaenus* mosquitoes, nine of which showed morphological variations typical of *Hg. leucophoebus* (Table). All specimens were photographed individually before molecular analysis.

**TABLE:** Overview of *Haemagogus leucocelaenus* specimen data

Sample ID	Municipality	Technique	Strata
513*	Rio Fortuna	Hand-net/oral aspirator	Canopy
514*	Rio Fortuna	Hand-net/oral aspirator	Canopy
1719*	Braço do Norte	CDC-LT	Canopy
1966*	São Martinho	CDC-LT	Canopy
2137*	São Martinho	Hand-net/oral aspirator	Ground
2138*	São Martinho	Hand-net/oral aspirator	Ground
2141*	São Martinho	Hand-net/oral aspirator	Ground
2210*	São Martinho	Hand-net/oral aspirator	Canopy
2212*	São Martinho	Hand-net/oral aspirator	Canopy
50	Santa Rosa de Lima	Hand-net/oral aspirator	Canopy
192	Santa Rosa de Lima	Hand-net/oral aspirator	Canopy
197	Santa Rosa de Lima	Hand-net/oral aspirator	Canopy
202	Santa Rosa de Lima	Hand-net/oral aspirator	Canopy
386	Rio Fortuna	Hand-net/oral aspirator	Canopy
713	Rio Fortuna	CDC-LT	Canopy
714	Rio Fortuna	CDC-LT	Canopy
927	Rio Fortuna	Hand-net/oral aspirator	Ground

Sample ID: identifier assigned to each specimen collected during the study; Municipality: city where each specimen was collected in Santa Catarina State; Technique: capture method used, including manual collection and CDC Light Trap with dry ice (CDC-LT); Strata: collection levels, categorised as canopy or ground. ^*^ *Haemagogus leucocelaenus* displaying morphological variations typical of *Hg. leucophoebus*.^7^ ^,^ ^8^

Genomic DNA was extracted from each specimen individually using the DNeasy Blood & Tissue Kit (69501 - Qiagen). The primers KUM07-F 5’ GGA TTT GGA AAT TGA TTA GTT CCT T 3’ and KUM07-R 5’ AAA AAT TTT AAT TCC AGT TGG AAC AGC 3’^16^ were used to amplify ~700 base pairs of the COI 5’ region, with the forward primer binding near position 200 in the *Hg. leucocelaenus* COI gene sequence (accession number MN531847.1), which is about 1,500 base pairs long. Polymerase chain reaction (PCR) was performed using an Applied Biosystems^®^ thermocycler under the following conditions: one cycle of 95ºC for 9 min, followed by 40 cycles of 95ºC for 30 s, 50ºC for 45 s, and 72ºC for 45 s, with a final cycle of 7 min at 72ºC. Positive amplicons were purified using the Wizard SV Gel and PCR Clean-Up System Kit (Promega) and sequenced (forward and reverse) using the ABI Prism 3730 DNA sequencer at the Oswaldo Cruz Institute and the ABI Prism Big Dye Terminator Cycle Sequencing Ready Reaction kit (Applied Biosystems, Foster City, USA).

The sequence quality was verified using CHROMAS version 2.4 software, and consensus sequences were assembled with SeqMan version 7.0. Molecular identification was performed using the National Centre for Biotechnology Information (NCBI) database. COI DNA sequence alignments were generated using ClustalX (https://www.ebi.ac.uk/Tools/msa/clustalo/). A phylogenetic tree was generated using IQ-Tree version 2.1.2^17^ with the Maximum Likelihood method and the best-fit substitution model TIM2+F+I. The resulting IQTREE file was then uploaded to the iTOL (Interactive Tree of Life) platform^18^ for visualisation. The analysis included *Hg. leucocelaenus* sequences from different regions of Brazil and Trinidad, with the following accession numbers: PP915664 (São Paulo), PP372854.1, MH118162.1, MH118163.1, PP372855.1, PP372856.1 (Sergipe), MN531847.1 (Pará), and MT987602 (Trinidad). Sequences from *Hg*. *spegazzinii* and *Hg*. *capricornii* (accession numbers MH118155.1 and PP915673.1, respectively) were used as outgroups.

## RESULTS AND DISCUSSION

During the study period, 91 females of the genus *Haemagogus* were captured across five municipalities in southern Santa Catarina, Brazil. The only species identified was *Hg. leucocelaenus*. However, morphological variations were noted in 22 specimens, leading to potential misidentification with *Hg. leucophoebus* (hereafter referred to as *Hg. leucocelaenus* ^*^ ). *Hg. leucocelaenus* specimens were collected as follows: Braço do Norte (38 mosquitoes), Pedras Grandes (2), Rio Fortuna (6), Santa Rosa de Lima (6), and São Martinho (17). Additionally, *Hg. leucocelaenus* ^*^ specimens showing morphological variations were found in Braço do Norte (6), Pedras Grandes (6), Rio Fortuna (2), and São Martinho (8).

**Fig. 2:**
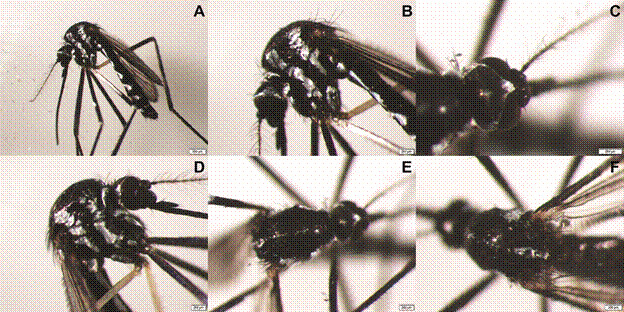
morphology of *Haemagogus* mosquitoes. (A) Lateral view of the whole mosquito. (B, D) Close-up lateral view highlighting the pleura with vertical bands of silvery scales. (C) Dorsal view of the head showing the vertex. (E) Dorsal view showing the scutum covered by dark scales, with acrostichal, prealar, and prescutellar lines or patches of silvery scales. (F) Dorsal view of the final portion of the scutum and the scutellum.^6^ ^,^ ^10^

*Morphological analysis* - All 91 identified *Haemagogus* specimens exhibited a scutum covered with dark scales, featuring acrostichal, prealar, and prescutellar lines or patches of silvery scales, as well as pleura with vertical lines of silvery scales (Fig. 2).

**Fig. 3:**
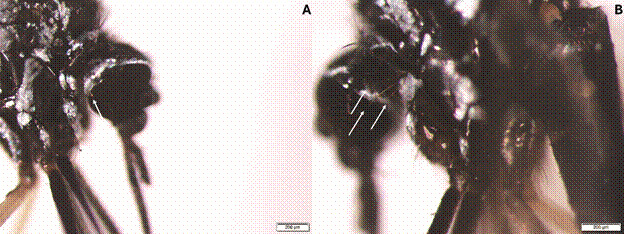
comparison of proepisternal setae in *Haemagogus* mosquitoes: (A) *Haemagogus leucocelaenus*, characterised by one long seta (indicated by the white arrow). (B) *Haemagogus leucocelaenus* ^*^ , exhibiting three long setae (indicated by the white arrows), a trait typical of *Hg. leucophoebus*.^6^ ^,^ ^10^

**Fig. 4:**
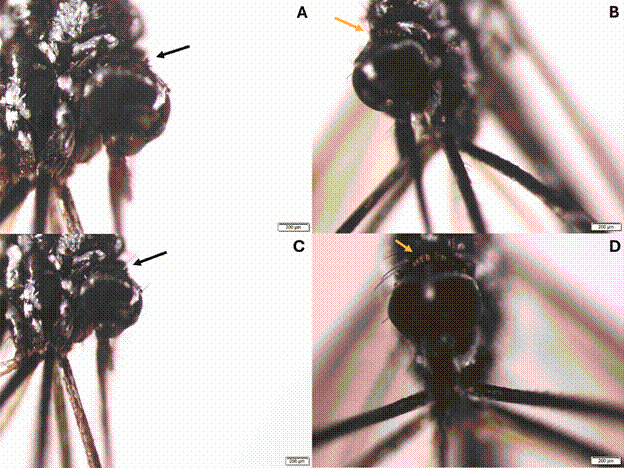
comparison of erect scales on the heads of *Haemagogus* mosquitoes: (A, C) *Haemagogus leucocelaenus* ^*^ , featuring erect scales that are entirely black (indicated by the black arrows), a trait typical of *Hg. leucophoebus*. (B, D) *Haemagogus leucocelaenus*, characterised by erect scales that are entirely pale brown (indicated by the yellow arrows).^6^ ^,^ ^10^

Notable morphological variations were observed in the *Hg. leucocelaenus* specimens: (i) Proepisternal setae differed, with *Hg. leucocelaenus* having one long seta, while *Hg. leucocelaenus* ^*^ specimens exhibited three, a trait typical of *Hg. leucophoebus* (Fig. 3);^6^ ^,^ ^10^ (ii) The erect scales on the head varied, with *Hg. leucocelaenus* ^*^ having scales that were entirely black or dark brown (characteristic of *Hg. leucophoebus*), whereas *Hg. leucocelaenus* had entirely pale brown scales (Fig. 4).^6^ ^,^ ^10^

Galindo et al.^11^ previously noted that distinguishing *Hg. leucocelaenus* from *Hg. leucophoebus* based on adult morphology is challenging due to reliance on structures or coloration that may be difficult to observe - factors that might have been overlooked in their study. Similarly, Zavortink^19^ reported variations in the colour of the erect scales on the occiput of *Hg. leucocelaenus* from Trinidad, Brazil, and Argentina, with Trinidad specimens showing dark brown scales akin to those of *Hg. leucophoebus* in Brazil.^11^ Marcondes and Alencar^12^ highlighted the importance of further research to explore potential regional morphological variations in *Hg. leucocelaenus*.

*Molecular analysis* - Molecular analyses were conducted on eight *Hg. leucocelaenus* and nine *Hg. leucocelaenus* ^*^ specimens (Table) to confirm the identity of *Hg. leucocelaenus* ^*^, which showed morphological variations typical of *Hg. leucophoebus*, a species previously identified only in Acre. The 5’ COI region of mitochondrial DNA was sequenced, with the resulting sequences (approximately 560 to 700 base pairs) submitted to GenBank under accession numbers PQ042427 to PQ042443.

**Fig. 5:**
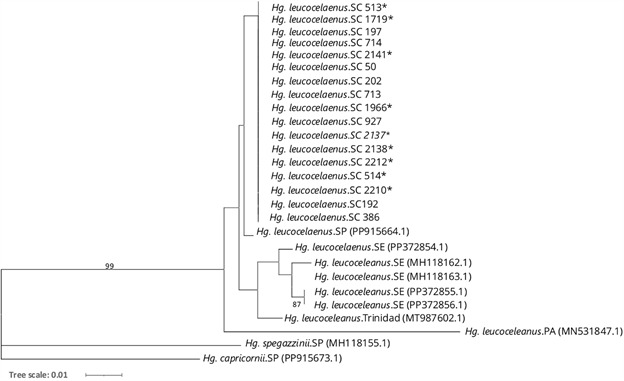
maximum likelihood tree based on Cytochrome C Oxidase Subunit I (COI) sequences (TIM2+F+I model). This analysis included 17 nucleotide sequences obtained in this study (*Hg. leucocelaenus*.SC), with sample IDs listed alongside the species name (see Table). Additionally, sequences from other regions of Brazil and Trinidad, obtained from GenBank, were included. *Haemagogus spegazzinii* and *Haemagogus capricornii* were used as outgroups (accession numbers provided in parentheses). This phylogenetic analysis strongly supports that all 17 *Hg. leucocelaenus* sequences from Santa Catarina belong to the same species, as they (i) exhibit a completely monomorphic COI genetic profile and (ii) form a monophyletic clade with *Hg. leucocelaenus* sequences from São Paulo, Sergipe, Pará, and Trinidad. Node values represent bootstrap percentages based on 1,000 replications, with only values above 75% displayed. ^*^ *Haemagogus leucocelaenus* displaying morphological variations typical of *Hg. leucophoebus*. SC: Santa Catarina; SP: São Paulo; SE: Sergipe; PA: Pará.

All 17 sequences exhibited a completely monomorphic COI genetic profile, with no observed nucleotide diversity. Molecular identification through the NCBI database revealed 95% similarity with *Hg. leucocelaenus* and 93% with *Hg. janthinomys*. The differences in similarity values can be explained by the origin of the *Hg. leucocelaenus* NCBI sequence, which is from Canaã dos Carajás, Pará, Brazil,^20^ 2,500 km from southern Santa Catarina. Mitochondrial DNA has a higher mutation rate than nuclear DNA,^21^ so significant differences can arise between mosquito populations from distant Brazilian regions. Despite this, the identical genetic sequences of all 17 specimens analysed in this study confirm that they belong to the same species. Additionally, the phylogenetic tree (Fig. 5) groups our sequences as a monophyletic clade with *Hg. leucocelaenus* sequences from São Paulo, Sergipe, Pará, and Trinidad, further indicating that all 17 mosquitoes analysed in this study belong to the *Hg. leucocelaenus* species.

Thus, despite the morphological differences, molecular identification confirms that both *Hg. leucocelaenus* and *Hg. leucocelaenus* ^*^ (which shows morphological variations typical of *Hg. leucophoebus*) are the same species. Morphological variations are also observed in other species. For example, Prudhomme et al.^22^ identified morphological differences in wing phenotypes in *Aedes albopictus* (Skuse, 1895), noting variations in shape and size. Similarly, Doorenweerd et al.^23^ showed that the morphological differences between *Bactrocera frauenfeldi* (Schiner, 1868) and *Bactrocera albistrigata* (Meijere, 1911) (mango fruit flies) represent intraspecific variation. Likewise, although differences in metasomal band colour visually separate *Xylocopa nigrocincta* (Smith, 1854) and *Xylocopa suspecta* (Moure & Camargo, 1988), Agostini et al.^24^ found no genetic differences in their COI sequences, suggesting they are not distinct evolutionary lineages. Thus, variations such as differences in the number of setae or the coloration of scales may simply reflect individual differences within the same species. Although COI has proven effective in identifying *Haemagogus* species,^25^ further studies using additional molecular markers are needed to confirm this hypothesis.

Recording and identifying the species responsible for YF transmission in epizootic areas is essential for effective disease control. This study is the first to report the presence of *Hg. leucocelaenus* in Santa Catarina, Brazil, and to provide DNA barcoding sequences for this species from southern Brazil. This is a key step in accurately identifying YFV vector species in South America.
